# Acute colonic pseudo-obstruction with bowel rupture after caesarean section in HELLP syndrome: a case report

**DOI:** 10.1186/s12884-020-03414-9

**Published:** 2020-11-25

**Authors:** Ying Peng, Cheng Peng

**Affiliations:** Department of Obstetrics and Gynaecology, First Affiliated Hospital of USTC, Division of Life Sciences and Medicine, University of Science and Technology of China, 17, Lu Jiang Road, Hefei, 230001 Anhui P. R. China

**Keywords:** Ogilvie syndrome, Caesarean section, HELLP syndrome

## Abstract

**Background:**

Ogilvie syndrome, also known as acute colonic pseudo-obstruction (ACPO), can occur postpartum after caesarean section (C-section), often resulting in caecal dilatation. The incidence rate is approximately 100 cases in 100,000 patients per year (Ross et al., Am Surg 82:102-11, 2016). Without proper diagnosis and treatment, it may progress to intestinal perforation or other fatal complications.

**Case presentation:**

A 39-year-old pregnant woman underwent emergency low-segment C-section due to complications of Haemolysis, Elevated Liver enzymes and Low Platelets syndrome (HELLP) syndrome. ACPO was suspected on the third day after C-section based on inability to pass flatus, evident abdominal distension, slight abdominal pain, and computed tomography (CT) scan revealing severe, diffuse colonic distention with caecal dilatation of approximately 9 cm. Based on these findings, conservative treatment was implemented. However, 6 days after C-section, her symptoms worsened, and CT showed possible intestinal perforation; thus, an emergency laparotomy was performed. Due to a 3-cm (diameter) laceration in the anterolateral wall of the ascending colon and a 5-cm tear in the ileocecal junction, in combination with mucosal eversion in the colon, resection of the ileocecum, distal closure of the ascending colon, and a terminal ileostomy were performed. The patient was discharged 2 weeks post-laparotomy and continued to undergo nursing care for the incision and stoma. Ileostomy was performed 4 months later.

**Conclusion:**

Ogilvie syndrome after C-section is an extremely rare but severe condition, which warrants early recognition and treatment to prevent potentially fatal complications, especially in patients with poor health status.

## Background

Acute colonic pseudo-obstruction (ACPO), also known as Ogilvie’s syndrome, is an organic disease caused by colonic dysfunction, which may be related to the inhibition of autonomic nervous function in the colon. It is mainly characterised by an acute and extensive expansion of the colon, mainly in the cecum and the right colon. The incidence of ACPO after caesarean section (C-section) is extremely low, making it a rare obstetric complication [1]. A study reported the postpartum incidence of ACPO to be 1:1460 deliveries [2], and another report estimated its prevalence to be 0.4% following C-section [3]. Its symptoms are easily obscured by postoperative wound pain and uterine contractions, and the specific mechanism underlying its manifestation remains unknown. Patients experience mild symptoms, such as abdominal pain, abdominal distention, and vomiting, which may worsen, leading to bowel necrosis, perforation, and colon torsion [4]. According to various reports, the risk of spontaneous colon perforation varies between 3 and 25%, and the associated mortality rate is as high as 50% [3, 5, 6]. Therefore, early recognition and management is crucial for improving prognosis [6, 7]. In this study, we report a case of HELLP with ACPO and colonic rupture after C-section.

## Case presentation

A 39-year-old pregnant woman, G1P0, with an irregular prenatal examination was hospitalised, under the description “37 weeks of gestation, 1 week of dizziness.” She had no significant medical history and was a non-smoker, with no history of constipation, drug abuse or accidental poisoning. Similarly, she has no history of mental illness or psychotropic drugs use. Notably, she had no significant family history. The patient had been unable to remain in a supine position at night since the seventh month of gestation, which was not given due consideration. The patient had been experiencing dizziness, vertigo, blurred vision, and oedema of both lower limbs for 1 week before being hospitalised, and her blood pressure measured 220/140 mmHg upon hospital admission. The patient was physically obese and weighed 125 kg (body mass index 44.8 kg/m2); she was hyperreflexic, and oedema was observed extending up to her sacral region. Results of related examinations performed after hospital admission were as follows: clear urine, urine protein level 3+, an elevated glutamic-pyruvic transaminase level at 362.70 IU/L, an elevated glutamic oxaloacetic transaminase level at 589.8 IU/L, plasma protein level of 66.90 g/L, decline in serum albumin level at 31.4 g/L, blood sugar concentration of 7.30 mmol/L, and decline in platelet count at 48 × 109/L; platelet count re-examination revealed 26 × 109/L. The patient underwent emergency low-segment C-section because of HELLP, followed by blood pressure control and platelet transfusion. Surgery was completed successfully and uneventfully with estimated blood loss of 300 ml. Following the prevention of eclampsia (by the administration of MgSO4), blood pressure control (by the administration anti-hypertensive agents), infection prevention, monitoring of vital signs, observation of postpartum haemorrhage, and regular check-ups, the biochemical parameters of the patient were normalised. The patient could not pass flatus 3 days after C-section and presented with evident abdominal distension, abdominal pain, normal electrolyte level, leucocyte count of 12 × 109/L, C-reactive protein level of 263 mg/L, and haemachrome decline at 72 g/L. An abdominal and contrast-enhanced pelvic CT scan revealed severe, diffuse colonic distention with caecal dilatation of approximately 9 cm. There was no evidence of mechanical obstruction or underlying gastrointestinal injury, suggesting ACPO. Conservative treatment, including fasting, gastrointestinal decompression, maintenance of electrolyte balance, and correction of anaemia, was implemented. A slight improvement was observed in the patient’s abdominal distension, and she could pass flatus and defecate. Two days after, the abdominal distension did not significantly improve; however, she could still pass flatus, and conservative treatment was continued. Unfortunately, her abdominal distension significantly increased 6 days after C-section, with no flatus and absence of bowel sounds. Moreover, electrolyte levels were normal, whereas heart rate increased to 130 beats/min; similarly, the leucocyte count increased, peaking at 16.33 × 109/L. Further, CT examination showed evidence of pneumoperitoneum; thus, colon perforation could not be excluded. A laparotomy was performed immediately after group consultation 6 days after C-section. Intra-operatively there were faecal-like material with present of ascites. Pus was noted covering the abdominal organs. The bowels were oedematous and dilated with a 3-cm hole noted at the antero-lateral wall of ascending colon. A 5-cm tear was also seen at the ileo-cecal junction with mucosal eversion. Therefore, resection of the ileocecum, distal closure of the ascending colon, and terminal ileostomy were performed. Intestinal tumours and mesenteric vascular occlusions were excluded as they were not identified during surgery. The patient was discharged 2 weeks post-laparotomy with no post-operative complications, and nursing care for incision and stoma was continued after discharge. The patient and her family members actively cooperated and agreed with the whole treatment process. Ileostomy closure was performed 4 months later. During the follow-up period, abdominal physical examination and abdominal CT, and colonoscopy were performed. No recurrence was observed at 2 years of follow-up.

## Discussion and conclusions

ACPO was first described in 1948 by Sir William Ogilvie. It is characterised by a functional colonic obstruction for reasons that remain poorly understood [4]. This syndrome has a mortality rate as high as 45%, and its signs and symptoms, if not rapidly recognised, may result in bowel perforation, faecal peritonitis, and death [5, 7]. Many conditions are associated with ACPO. Common risk factors for severe complications or infections, including critical illness, recent hand surgery, metabolic imbalance, and other non-surgical causes [3, 6, 8], in elderly patients and inpatients, are widely reported [5, 9, 10]. In women, the most common causes are C-section and pregnancy, followed by pelvic surgery and trauma [11, 12]. Similarly, Clayman et al. suggested that C-section was the most common cause, followed by urologic surgery [13].

The occurrence of ACPO after C-section is related to trauma, anaesthesia, or drug use affecting the autonomic nervous system; however, the exact mechanism is unclear. Pathogenic factors include long delivery time, maternal fatigue, maternal weight loss, poor general health status, fluid loss, hypoalbuminemia, postoperative analgesia, and electrolyte imbalance or acid-base balance disorder [14]. High progesterone, glucagon, and prostaglandin levels during pregnancy are similarly considered possible risk factors for ACPO in obstetric patients [15]. ACPO may be induced in combination with acute physiological disorders, such as preeclampsia and postpartum haemorrhage. A recent systematic study showed that patients with preeclampsia, multiple pregnancies, postpartum haemorrhage, or placenta praevia are more likely to develop ACPO after C-section [16]. Our case was accompanied by HELLP syndrome, with hyperproteinaemia, hypertension, elevated liver enzymes, and thrombocytopenia.

An accurate diagnosis is still difficult [5, 17, 18]. ACPO symptoms generally manifest within 48 h after surgery and within 12 days at the longest [19]. Abdominal distention and tenderness were the most common symptoms, followed by vomiting. The abdominal X-ray plain film or CT examination mainly showed the expansion of the cecum and the ascending colon. Evident abdominal tenderness, fever, leucocytosis, and caecal dilatation > 12 cm are factors that may indicate colonic ischemia or perforation [3, 5].

The treatment for ACPO is essentially colonic decompression. Conservative treatment is preferred, while laparotomy is contraindicated in the absence of peritonitis [20]. If continuous examination and abdominal X-ray do not indicate colonic ischemia, perforation, or impending perforation, it can be treated conservatively for several days. The success rate of conservative treatment ranges between 77 and 96%, and remission is usually within 3–5 days [3, 21, 22]. It involves avoiding opioids and other drugs to inhibit intestinal peristalsis, a correction of hyperproteinaemia, electrolyte disorder, and acid-base imbalance, continuation of gastrointestinal decompression, administration of barium enemas, treatment of other potential diseases or infection [5, 7, 23, 24], and the use of neostigmine if necessary [10]. Neostigmine is an anti-cholinesterase inhibitor, and its therapeutic purpose is to improve the colonic peristalsis caused by sympathetic stimulation or parasympathetic dysfunction. Because of the risk of bradycardia, its use should be closely monitored and corrected with atropine if necessary [25]. Barium enemas can not only alleviate the disease but also indicate colon expansion and are helpful to eliminate mechanical intestinal obstruction. However, it increases the risk of intestinal perforation. Surprisingly, it has been suggested that chewing gum can promote the recovery of intestinal function [26], and irritant laxatives, which can aggravate colonic distention, should be avoided [27]. Bargiela et al. reported a case of chronic obstructive pulmonary disease (COPD) symptom relief after treatment with a potassium-sparing agent [28]. If the cecum’s diameter is > 9 cm, it can be decompressed by colonoscopy if the conservative treatment is ineffective [29]. Some consider colonoscopy better than treatment with neostigmine and even suggested it as a first-line treatment [30]. This is because the success rates of colonoscopy decompression and neostigmine treatments were 74 and 29%, respectively. Nonetheless, about 10% of patients experience a certain degree of colon ischemia during colonoscopy; therefore, indications should be strictly controlled, considering a perforation rate of 1–3% [9, 31].

Every 12–24 h, it is recommended to monitor vital signs and the abdominal plain film and perform continuous abdominal examination [32], ACPO’s early sign is anal exhaust or defecation. However, defecation does not represent the recovery of normal intestinal function, and some patients still have a chance of experiencing further deterioration. Imaging- and abdominal examinations have important guiding significance for disease prognosis [33].

If the conservative treatment is ineffective, timely surgical treatment is needed. Surgical treatment need is mainly observed in unresponsive symptoms or progressive aggravation, especially when the cecum diameter is > 12 cm or the abdominal distention lasts for more than 6 days and there is peritoneal irritation or air under the diaphragm [3, 22]. According to Laplace’s law, the cecum diameter is the most important, because it is the most likely area for perforation to occur. Some studies have shown the need for surgical treatment when the cecum diameter is > 9 cm and is accompanied by intestinal ischemia [34]. When the cecum diameter is > 14 cm [3], the incidence of complications is 23%. According to a retrospective analysis of 66 patients, intestinal perforation occurred in all patients whose cecum diameter was > 12 cm, and in 3 of 17 patients whose cecum diameter was < 9 cm, most of the perforation occurred from the third day to the fifth day. If the cecum diameter is > 12 cm and dilation time is more than 6 days, the incidence of colon necrosis or perforation is 3–10% [35]. An analysis of 221 patients with ACPO showed that the ACPO mortality rate was 15%. When the cecum diameter was > 14 cm, the mortality rate doubled, and the decompression delay was more than 7 days. In comparison to the 4-day delay, the mortality rate increased 4 times [2]. The risk factors for death from ACPO were old age (> 60 years old), caecal diameter > 14 cm, and long duration of colonic dilatation (> 4 days) [3]. However, some studies have similarly demonstrated that for emergency surgery, it depends more on the urgency of the patient’s symptoms than the colon diameter. Many patients have dimensions larger than this value (12 cm for the cecum) without sequelae. The acuteness of the disease and the duration of continuous expansion may affect the risk of perforation [36].

Surgical methods include colostomy, repair of intestinal perforations, or colectomy, and the specific surgical methods are selected based on the presence of colon necrosis and the general patient situation. The success rate varies from 95 to 100%. At present, there is no comparative data to suggest colostomy as first-line therapy [3, 18].

In summary, our study findings suggest that ACPO after C-section in HELLP syndrome is a rare and severe postoperative complication, which should be identified and treated as early as possible. The management of ACPO requires a multidisciplinary approach for a better outcome.

## Data Availability

The data sets used and analysed during the current study are available from the corresponding author upon reasonable request.

## Abbreviations

ACPO: Acute colonic pseudo-obstruction
HELLP syndrome: Haemolysis, Elevated Liver enzymes, and Low Platelets syndrome
C-section: Caesarean section
COPD: Chronic obstructive pulmonary disease
